# The role of flow injection analysis within the framework of an automated laboratory

**DOI:** 10.1155/S1463924690000116

**Published:** 1990

**Authors:** Peter B. Stockwell

**Affiliations:** P S Analytical Ltd Arthur House B4 Chaucer Business Park Watery Lane, Kernsing, Kent Sevenoaks TN15 6QY UK

## Abstract

Flow Injection Analysis (FIA) was invented at roughly the same time by two quite dissimilar research groups [1,2]. FIA was patented by both groups in 1974; a year also marked by the publication of the first book on automatic chemical analysis [3]. This book was a major undertaking for its authors and it is hoped that it has added to the knowledge of those analysts attempting to automate their work or to increase the level of computerization/automation and thus reduce staffing commitments. This review discusses the role of FIA in laboratory automation, the advantages and disadvantages of the FIA approach, and the part it could play in future developments.

It is important to stress at the outset that the FIA approach is all too often closely paralleled with convention al continuous flow analysis (CFA). This is a mistake for many reasons, none the least of which because of the considerable success of the CFA approach in contrast to the present lack of penetration in the commercial market-place of FIA instrumentation.


Journal of Automatic Chemistry, Vol. 12, No. 3 (May–June 1990), pp. 95–103

### The role of flow injection analysis within the framework of an automated laboratory

**Peter B. Stockwell**
*P S Analytical Ltd, Arthur House, B4 Chaucer Business Park, Watery Lane, Kemsing, Sevenoaks, Kent TN15 6QY, UK*

*Flow Injection Analysis (FIA) was invented at roughly the same time by two quite dissimilar research groups [1,2]. FIA was patented by both groups in 1974; a year also marked by the publication of the first book on automatic chemical analysis [3]. This book was a major undertaking for its authors and it is hoped that it has added to the knowledge of those analysts attempting to automate their work or to increase the level of computerization/automation and thus reduce staffing commitments. This review discusses the role of FIA in laboratory automation, the advantages and disadvantages of the FIA approach, and the part it could play in future developments.*

*It is important to stress at the outset that the FIA approach is all too often closely paralleled with convention al continuous flow analysis (CFA). This is a mistake for many reasons, none the least of which because of the considerable success of the CFA approach in contrast to the present lack of penetration in the commercial market-place of FIA instrumentation.*

### Introduction

Automatic methods of analysis have become well established, and automated analysers, either commercial, or constructed in-house, have become common features in most analytical laboratories. In clinical laboratories the advent of automated analysers has eased the considerable bottleneck caused by increased efforts in health care; and in process control, automated analysers, both on-line and off-line, have been used with considerable success: the former greatly modifying the work pattern in the laboratory and the latter greatly increasing the speed of response to system changes. Industrial laboratories have not had as much success as clinical laboratories in automation and this is generally considered to be because of the complexity of the sample matrix; and, it must also be said, to a resistance to change or to an inability to look at analytical problems in different ways.

There has so far been a notable resistance from universities and colleges to include any aspects of automation in their syllabuses. This, however, is changing with the introduction of FIA and the use of microcomputers.

FIA techniques are both inexpensive and 'easy to teach', and the latter represents the brave new world of electronics (rather cynically one might say it is the ability to repeat work with the addition of a microcomputer).

The continuing favouring of these techniques, without a clear understanding of the role of automation in the practical world of analytical chemistry, has tended to restrict rather than advance the progress, particularly for FIA (this situation will be discussed in full later).

Despite these problems, there is a well-established commercial market for automatic instrumentation. Over the last few years, technological progress, in terms of equipment design and increased ranges of applicability, has been exceptional. A systems designer, working in a large instrument company, in a small business concern, or in a laboratory, has a wide range of modules and resources available, so it is possible to configure a fully automated system which is well constructed and designed, and which will operate reliably for a long time. This latter point is of vital importance in terms of acceptability to the user. The introduction of FIA into this scenario has been restricted, in my view, because it has been presented by some of its advocates more as a toy than as a real analytical tool, which can cope with the needs of a routine analytical laboratory.

Automatic analysers must be justified in terms of increased efficiency of laboratory operations, increased capacity and throughput of samples, and, most importantly, in terms of cost effectiveness. This latter aspect is very complex but any automation under consideration must be scrutinized on economic grounds prior to acceptance. The principal concern of laboratory managers should be to ensure that the criteria for automation are met, and that the automation installed provides for effective control and management.

Automation, by its very nature, requires a fundamental change in the role of the analytical chemist. In a manual approach, the analyst controls the entire procedure, even if highly sophisticated instrumental measurement systems are being used. This personal control leads to pride in the quality of work and confidence in the results. When the same analytical chemist is operating an automated analyser capable of analysing samples at a high rate, the analyst's involvement, particularly when the equipment is functioning correctly, is limited to loading samples and reading results. It can be very easy for an analyst to feel degraded by this approach and to avoid this it is sensible to use the analyst to evaluate new methods, and for less well qualified staff to run the automatic systems. If these are well designed, particularly with full verification of methods and operation, the staff will feel confident that they are carrying out the procedures to the letter and the analytical results will be very reliable. Rather than degrading the analyst's role, it assures greater importance. The senior management, however, has to change its attitudes to retraining and researching new developments to maximize the effective use of staff.

Despite the impact of automation on laboratories, there is remarkably little in the way of texts on the subject. However, a series of journals have been produced over the last few years, which cover some areas of laboratory automation [4,5]. Automation extends beyond the simple instrumentation aspects, and transgresses into management techniques, so it is difficult to look at it in terms of a single scientific subject area. Automation also crosses disciplinary boundaries, and clinical, industrial and process chemists have much to gain from each other's experience of automation in routine use. Unfortunately, one of these journals has not been able to continue and the others require more active support from instrument companies and from the analysts themselves to increase their frequency, and therefore speed, of publication.

Recently, a new book has been published by Valcarel [6] which provides an excellent contribution to the field. Its emphasis is perhaps on the academic, rather than industrial, side but none the less it contains valuable detail.

### What is automatic analysis?

'Automatic Analysis' is well established as a subject area but less well defined in terms of what that area embraces. The International Union of Pure and Applied Chemistry (IUPAC) defines automation as 'the use of combinations of mechanical and instrumental devices to replace, refine, extend, or supplement human effort and facilities in the performance of a given process, in which at least one major operation is controlled without human intervention, by a feedback mechanism' and mechanization is defined as 'the use of mechanical devices to replace, refine, extend or supplement human effort'. The distinction between the two terms is clear; IUPAC recommends that 'automation' be reserved for those systems involving feedback loops. This is logically sound but, like many other definitions which have followed the growth of a subject, it is likely to take some years before this distinction is adopted in common usage. The motivation for research and development in automation and/or mechanization was largely twofold; to improve the cost-effectiveness in discharging large analytical workloads, especially those of a repetitive nature, and to improve method performance, notably precision, in these circumstances. The term 'automation' has been commonly used to describe the advances made. In this review, the term 'automation' includes all those developments which have minimized human intervention in chemical analysis and thus released the analyst for more demanding tasks.

The principal operations involved in a chemical analysis are listed in the table.

In considering the potential benefits of automating an analysis, each item in the table should be considered, although in many instances, sampling will be an external operation which is unsuitable for automation. The majority of publications in the field of automatic analysis have concentrated on particular parts of the total subject as represented in the table. The sample measurement stage has received the most attention, and almost all the measurement techniques used in chemical analysis have been partly, or fully, automated. More recently, attention has been focused on the post-analysis stages, and data processing using computers, microprocessors, or simple calculators, has been extensively studied. It is now cost-effective to use simple personal computers to control automation, calculate results, and then prepare reports in a suitable format, before communicating with a central computing facility. However, by far the most demanding stages of the analytical system are sampling and sample preparation and little work has so far been done in these areas. The most significant efforts to date in these fields have been made by Professor Knapp and his team in Graz, Austria and at the Laboratory of the Government Chemist in London. The results of the former research are available commercially from Anton Paar Graz Austria. The latter research is described in detail by Stockwell and Foreman [7].

*Components of a chemical analysis.*

| |
|---|
| (1) Sampling. |
| (2) Sample pretreatment. |
| (3) Sample measurement, including standardization and calibration. |
| (4) Control of instrument parameters. |
| (5) Calculation of results, initially the analytical result and then for the sample itself. |
| (6) Report generation, distribution and archiving. |

In planning the introduction of automation, it is important to consider all the stages of the analysis, as well as the function of the laboratory within its institution or business. Thus priorities will be properly identified; these could include, for example, the need to collate results of multiparameter analyses before reporting, and, in clinical analysis, the preservation of sample-patient identification.

Research into automation of analytical techniques is prompted by the need to produce cost-effective solutions to an ever-increasing demand for chemical analysis. The demand comes from increased legislative control in many areas, as well as a growing concern for the quality of the environment, both in general and in the workplace.

In this respect the analytical chemist can provide a valuable input by providing facilities for good quality measurements but the public must bear the costs involved. Automation and mechanization are clearly consistent with these needs: in order to satisfy demand, adequate techniques to automate sample handling, measuring and reporting of the results, must be devised.

The limiting factor in the development of automatic analysis is rarely the development of analytical techniques. The need for long-term operation places considerable constraints on the choice of materials and components used in the construction of automatic instruments, particularly moving parts. Often, however, it is possible, with the correct specification of the analytical requirements, to select analytical techniques to avoid the use of solvents and aggressive reagents and thus minimize damage.

Little performance data has been published on routine automatic instruments; even in clinical areas it has often been difficult to obtain proper inter-laboratory assessments of instruments.

Exceptions to the above comments can be found in earlier issues of the *Journal of Automatic Chemistry*. It is the intention of this journal to serve as a medium to communicate the problems as well as the merits of automation. The impact of automation extends beyond the instrumentation and influences such factors as management style and choice of staff. All these areas of interest are valid items to publish in the journal.

The rate of development of 'new' instruments far exceeds the accumulation of meaningful performance characteristics on existing ones. To some extent, this situation has arisen because there is no international committee or organization to co-ordinate efforts – this is perhaps not surprising in view of the breadth of the subject.

The circumstances in which an instrument might be operated can be extremely varied. It may be used, for example, by non-scientific staff to control a process plant in a hostile environment. The design criteria for instruments of this type are, of necessity, far more demanding than for those used in a clean laboratory by a graduate scientist. Further restrictions to progress in the use of automation are largely economic. It is a fact that commercial companies largely define market areas, so that the development and marketing costs are spread. In the clinical area this has been less limiting since the analyses and sample matrices are not as varied as in the industrial sector.

If the analytical requirements in a laboratory cannot be met by commercial products then a system must be developed in-house. Design and development of a single custom-built instrument can be extremely expensive, and it is difficult to recover development costs during the operating lifetime of the instrument. Automatic analysis is therefore a widely diverse and complex area of instrumentation; FIA is one of those newer techniques which is attempting to become accepted in routine long-term use.

### Economic aspects of automation

The reasons for installing analysers to replace manual methods are many and varied, and priorities will be related to the nature of the organization of which the analytical laboratory is a part. Nevertheless, cost-effectiveness is a major motivation, and this can be assessed, both as an internal laboratory study or, in the industrial environment, for the company as a whole. In the latter case, automatic analysis can improve efficiency by reducing, or eliminating, the need to store intermediates or the final product; and the financial advantage so gained is likely, in most circumstances, to far exceed the cost of automatic equipment. Where the economic case for automation must be made internally, which would be so for a consulting analytical laboratory, the true cost of manual and automatic analysis must be calculated to determine whether the additional output, or staff savings from automatic analysis, will outweigh the capital cost of the automatic equipment over a reasonable period.

To a first approximation, these costs can be compared in the following way. The cost of an analysis performed manually ($C_{man}$) can be expressed as:

$$C_{man} = F_{man}\,(T_s A_s + T_p A_p + T_m A_m + T_c A_c) \quad (1)$$

where $T_s$, $T_p$, $T_m$ and $T_c$ represent the time taken for sampling, pretreatment (physical or chemical) of the sample, measurement, and calculation and reporting of the sample respectively. The $A$ factors for each stage represent the proportion of the operator's time needed (for ashing, drying, and incubation it is very small). $F_{man}$ is a tariff factor, incorporating staff and overhead costs to convert the analytical time to a true cost. For the same analysis carried out fully automatically the cost $C_{aut}$ is given by:

$$C_{aut} = F_{aut}\,(T_{aut} A_{aut} + T_{maint} A_{maint}) \quad .. \quad (2)$$

where $T_{aut}$ and $T_{maint}$ are the times for automatic analysis and equipment maintenance, $A_{aut}$ and $A_{maint}$ are the proportion of operator time required for analysis and maintenance, and $F_{aut}$ is the cash conversion factor. If the method is not fully automated, any stage which remains manual can be costed as in equation (1) and carried into equation (2).

In those cases where the automatic method is faster than the manual one, or where the operator can perform more analyses in a given period, the cost advantage can be calculated. Even when there is little or no difference in time of analysis between the manual and automatic methods, equations (1) and (2) show that significant savings in operator time can accrue if the $A$ factors can be reduced to very small values in the automatic method; this should be the case for a well designed and reliable automated method.

An economic advantage of automation which falls outside the scope of the above equations is the potential 'silent hours' use of a proven automatic method. Thus a further two-to-threefold increase in throughput for the analysis under evaluation is potentially available, and this has particular appeal to a laboratory wishing to increase its analytical capacity without increasing its staffing. However, for 'silent hours' working several factors must be carefully evaluated. Safety in operation becomes paramount Ruggedness of the chemistry of the method is also important. Fluctuations in line voltage and ambient temperatures can occur overnight, and if the method is sensitive to such effects then incorrect results can occur even when the instrument is working satisfactorily. The analytical chemist is often the only person aware of these problems and this is another example of the professional's changed role as more automation is introduced.

For the automatic method to be preferable purely on economic grounds, the cost of analysers must be less than the manual cost by at least an amount equal to the cost of the automatic equipment amortized over a period of 3 to 5 years. For many of the more expensive instruments, particularly those in the clinical market, leasing agreement are common; in these cases the actual cost will be less for the automated regime. However, this simple mathematical treatment is very approximate and takes no account of the differences in reagent costs, power requirements and supervisory expenses between the two regimes.

In automatic continuous flow analysers, the reagent costs may well be significant. However, in many situations, suitable modifications of the chemistry or improvements in design, such as transfer from Technicon AA1 to AA2 methodology, can produce savings in reagent costs. This has two advantages; it reduces the reagent consumption and the analysis time. FIA has these advantages – the time scale of the analysis and the reagent consumption are both reduced.

However, this comparison is often a problem in as much as the chemistry suitable for FIA is unlikely to be similar since the rate of analyses are significantly different. Nonetheless, the potential advantages of time and reagent costs are clearly applicable in respect to the simple equation put forward here. To some extent the treatment described above assumes that the analytical procedure remains common throughout the two regimes. This may not be true. Norris and Hart [8] have introduced the concept of infrared reflectance spectroscopy in the analysis of moisture, oil and fat in cereal products. The approach is available commercially – an example is the Technicon Infra-Analyzer. For the analysis of grain, a series of chemical procedures is replaced by a single measurement of six infrared regions and reference to a suitable computer calibration. The transfer to automation in such a case is compelling in that it yields advantages in reagent costs and speed analysis. The development of wavelength scanning systems by L T Industries Inc. (6110 Executive Boulevard, Rockville, Maryland 220852, USA), Pacific Scientific (2431 Linden Lane, Silver Spring, Maryland 20910, USA) and Guided Wave International, (Karbingatan 22, 252 66 Heslingborg, Sweden) has extended the scope of the new infrared technique. Such systems have the major advantage of removing, or at least significantly reducing, the sample handling burden.

The economic treatment is also limited to an analytical chemical laboratory and it is valid where samples are received from an outside source but it is unlikely to be true where the laboratory is an adjunct to a processing plant and is performing quality-control analyses on to the plant operation. The cost of the automatic equipment will be small in relation to the plant costs and it will be improved precision of analysis and speed of response that will therefore have the greatest economic significance.

The installation of automatic analyses in the production line is an ideal in quality control, and there is ample scope here for additional automation. In these applications the speed and simplicity of FIA offers significant potential advantages which should be vigorously explored. However, this situation is one where the arbitrary boundaries introduced into analytical chemistry have hindered progress. Too often the chemists concerned are insular in their approach and have little, if any, interaction with automatic chemistry in related environments and industries. There is a clear need for these groups to broaden their horizons and attempt to transfer technology from related areas to solve problems in their own industries.

Care must be taken in estimating the cost of changing to automatic analysis, because the effects of so doing are far-reaching. There is a substantial impact on the role of the analytical chemist and on laboratory management and organization. To become fully conversant with an automatic analyser requires an understanding of the principles underlying analyser design, electronics, mechanics and data processing.

While a relatively unskilled operator can run an instrument, it is necessary to have more highly qualified staff available to detect, diagnose and rectify faults. The introduction of automation on an appreciable scale in a laboratory, which has hitherto depended on manual analysis, is almost certain to call for adjustment of staff expertise, with an increased emphasis on non-chemical support disciplines.

Whether an automated system is being designed in-house, or a high-cost instrument is being purchased, a detailed consideration of the costs involved is a valuable exercise. The procedure outlined here has proved to be valuable in a number of situations, and if the result of the analysis is a move to a different manual technique, this is still a positive benefit.

### Advantages of automation

The economic advantages which can accrue from the automation of an analytical procedure have been set out in the previous section. Attention is devoted here to the additional benefits which automation can produce. These are largely scientific in character and relate to improvements in the quality of results and the potential increase in the range of analytical methods which can be used. To derive full benefit from the introduction of automatic equipment, it is essential that proper consideration is given, at the outset, to making sure that the analytical equipment installed matches the analytical needs as closely as possible. This is an important new role for the analytical chemist and one for which laboratory management, manufacturers and educational institutions, must develop principles for guidance. Specification is of fundamental importance where automatic analysers are to be designed and constructed, rather than purchased from commercial suppliers. Over-design is expensive and time consuming; under-design can mean that the product will not meet the full analytical requirements. Specification includes the chemical procedure to be used. Direct conversion of a manual method includes steps, such as precipitation, which do not conveniently lend themselves to automation. In such instances, it is often preferable to modify the method to produce the full economic and scientific benefits of automation. Significant advantages can be gained from this type of study, even if the result is that new manual procedures are introduced rather than automatic ones.

If proper attention is given to calibration and standardization, a well-designed, constructed and maintained automatic analyser will operate reproducibly over long periods in the hands of a trained operator. It may therefore be expected that for a large batch of samples the analytical precision will be superior to that obtainable by manual analysers; the automatic method eliminates human error and fatigue, both of which are likely to become important as the sample size of the batch increases.

Of prime importance for automatic sample analysis is the reproducibility of the timing sequences. This enables reaction conditions to be carefully controlled and leads to improved performance when the method includes stages in which reactions do not proceed to completion, or where the analyte being measured is unstable. Separation techniques like dialysis and solvent extraction, in which the recovery of the desired species is frequently incomplete, can give highly reproducible automatic performance when accurately sequenced, and it is possible to employ colorimetric reactions where the colour stability would be inadequate for manual analysers. There are advantages here in the FIA approach – the range of potentially useful analytical reactions can be increased, provided that suitable standards, processed in exactly the same manner as samples, are available. A further extension is possible where an automatic analysis is carried out in a closed system; materials which are toxic or unstable in air can be more conveniently analysed than by a manual method.

Automation has proved to be an important factor in the rapid development of reaction-rate methods of analysis in which the analysis is made over a very small fraction of the total reaction time.

The ability to control operating parameters precisely in an automated technique can enable an exacting analysis to be carried out automatically in circumstances where the manual approach is extremely difficult. This is well illustrated by reference to the determination of very low concentrations of selenium in water. The manual method, based on automatic absorption spectrophotometry after conversion of selenium to its hydride, performed too imprecisely at the concentrations encountered. An automated procedure based on continuous flow, and using the same analytical method, gave much improved performance. The automated procedure was based on work of Goulden and Brookbank [9] and was further developed by Porter and Dennis [10]. Advantage is taken of controlled sequencing, but additional modifications were made to further improve the stability of the system. Several commercial hydride generators have been based on this work [11–13]. An evaluation of one of these shows the low levels of detection obtainable, as well as the excellent precision [14].

Well specified and designed automation improves the analyst's overall control, both in the quality of measurements and the value of results. Many analysts fear that by employing automatic techniques, and particularly computers, they will lose all control and that incorrect decisions will then be made. The analyst's vital role has already been briefly considered in this article; any automatic scheme should involve the analyst in all stages of planning and implementation. Freedom from the mundane chores inherent in manual methods should enable the analyst to carry out the required degree of quality control and to program and execute repeat analyses when required.

Coupling with computers will facilitate the appropriate feedback of information for improved control. In this respect the FIA approach offers the possibility of on-line method optimization. Betteridge *et al.* [15] and more recently Wentzell *et al.* [16] have described a versatile system using FIA principles which will optimize method conditions using simplex optimization strategies.

### Limitations of automatic analysis

Automation has many virtues; it also has some problems. In almost all the automatic procedures, performance is related to the analyses of standard materials of similar compositions to the sample. Often it is difficult to obtain standards having similar matrix effects to the samples under test. This can become a considerable limitation. However, in some applications it can be overcome by the skill of the analyst in devising new methods of analysis.

Although the introduction of automated analysis has improved the capacity and performance of many laboratories, some obvious limitations remain. In hardware terms these relate principally to restrictions imposed by design and by the materials used in construction, the ideal situation, in which any manual method may be automated where the demand exists, is far from being achieved. While almost every analytical measurement technique is amenable to some degree of automation, this is not so for a number of pretreatment and separation techniques. Precipitation and filtration are difficult to incorporate into an automatic system. Although several commercial automatic analysers offer filtration and precipitation facilities, a generalized, rugged design has not yet been marketed. Separation techniques, notably solvent extraction, distillation, and chromatography, can now be carried out successfully as part of an automated procedure, even in FIA systems, but they are limited to closely defined conditions of operation, and they lack the flexibility that a skilled analyst can achieve.

It must be remembered that the analysis of large numbers of essentially similar samples provided the initial economic impetus for the development of automatic techniques, and that by the use of a variety of design criteria, this challenge has largely been met.

However, the success achieved, particularly in respect of quality of results and financial benefits, has inevitably led to a demand for the design of automatic analysers offering increased flexibility, i.e. choice of method, nature and quality of reagents added and of time sequences, and also for the provision of procedural options available by command control. Clearly such requirements constitute a major focus for the generation of automatic analysers now being developed.

The need to perform analyses in vessels made of materials which are inert to the analytical medium is a fundamental one, but it becomes especially demanding where many samples are to be processed through the same vessels. The limitations of materials of construction for pumping tubes for continuous flow automation, are well known, as are the means, albeit inconvenient, of overcoming them by techniques like displacement pumping [17]. The design of an automated wet oxidation system [18] for the preparation of extracts for trace metal analysis, highlights many problems of pumping corrosive fluids.

Design capability and availability of appropriate materials of construction both contribute to a vitally important requirement of automatic analysers, that of reliability in service; loss of working time due to breakdown represents a serious limitation in achieving the basic requirement of correct and cost-effective operation. Although there is now a wealth of design and operational experience, there are, as yet, few critical evaluations published on component or module performance. Both users and designers would benefit from validated data on component performance.

Because the requirements for automation are much influenced by the role of the laboratory in which it is to be applied, the specification of any requirement is best carried out in-house. To do this, suitably qualified and experienced staff must be available, and this places a limitation on the extent to which automation can be considered in some laboratories. In many industrial situations the problems are so specific that commercial manufacturers are reluctant to use development funds to solve them because it is unlikely that a product line with more than a few sales will result. Consequently, in-house solutions must be sought, or the requirements contracted out against an adequate specification. This is expensive and it is a major limitation in automated system design.

It is important to recognize the limitations of automatic instrumentation, but with such a wide range of techniques available and with recent developments in electronics and computing, almost any analytical requirement can be automated. The main problems to be overcome are related to a proper understanding of the chemistry involved and the provision of materials and devices that are inert to the chemicals used in, or generated by, the analytical procedure.

### Recent developments in automatic analysis: chip technology

The various developments in the philosophies adopted to automated analysis have been previously reviewed by the author [19]. More recently, however, there has been a significant investment in chip technology: 'chips with everything'. The most significant advantage is that the chip technology has (*a*) enabled a series of array detectors to be developed giving multi-wavelength data instantaneously; and (*b*) enabled significant computing power to be added to such instruments as infrared reflectance spectrometers. In the latter device, significant amounts of data can be collected and correlated using mathematical principles to discern the significant wavelengths. Analysis of grain, tobacco, sugars and many other samples can be carried out (without any significant sample preparation) by analysis of the reflectance spectra correlated against normal analytical data. A further aspect of chip technology has been developed by two commercial companies for the analysis of clinical samples, one using photographic chemistry and the other the absorbent layers originally developed for urine analysis.

The salient principles of the Kodak *photographic style* system have been published by Curme *et al.* [20] and by Spayd *et al.* [21] and performance trials and evaluation tests on the technique indicate that it is both reliable and accurate. An evaluation has been published by Haeckel *et al.* [22], and, more recently, the alternative system due to Ames has been critically reviewed by Clark and Broughton [23]. The chip concept has also been applied to FIA. Recently, Ruzicka [24] described a logical extension to the flow pump manifold designed as a system of integrated conduits situated in a permanent rigid and planar structure. The grooves forming the flow channels may be imprinted or engraved into a transparent plate and then closed by a flat layer section. The rigidity of the structure ensures repeatability of the dispersion in the sample zone, thereby providing further miniaturization of the FIA system.

A further step involves integrating the sample introduction and the detection into one micro conduit, thus forming a micro-chemico electronic device. Fibre optics are used to interface these units to conventional spectrophotometers. The size and cost of these systems will open up new fields of application for FIA and transfer the testing from laboratory bench to being on-site.

### Flow injection analysis in perspective

The considerable number of publications of FIA are evidence of its rapid growth and wide applicability. Since the initial work in 1975, the developments have been spectacular, leading to more than 600 publications,

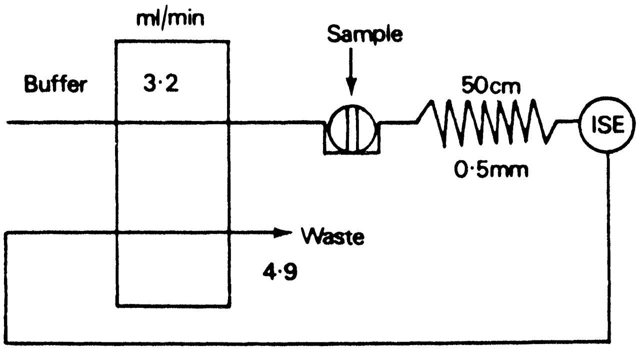

*Figure 1. Schematic diagram of simple flow injection system.*

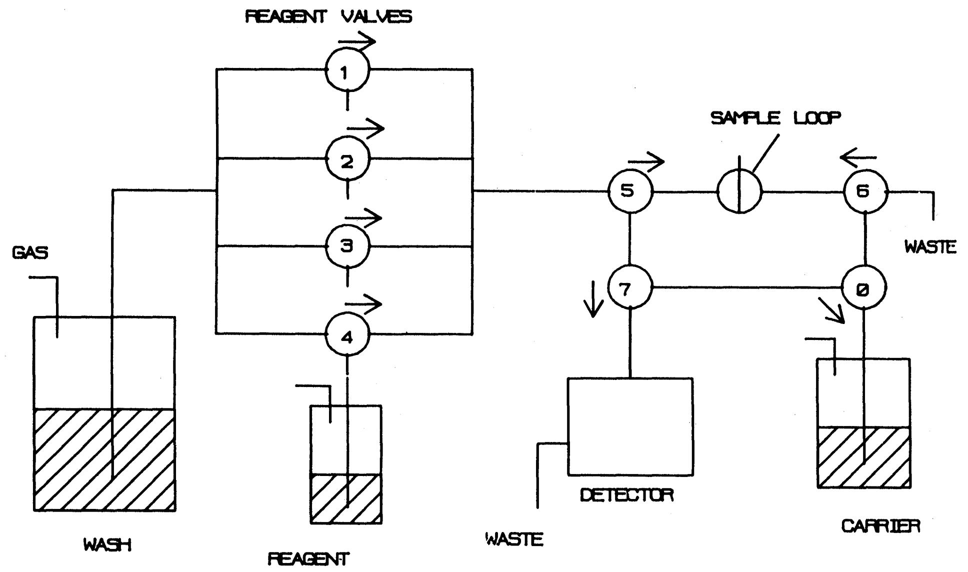

*Figure 2. Schematic diagram of the novel flow injection manifold described by Malcolme-Lawes* et al. *[26].*

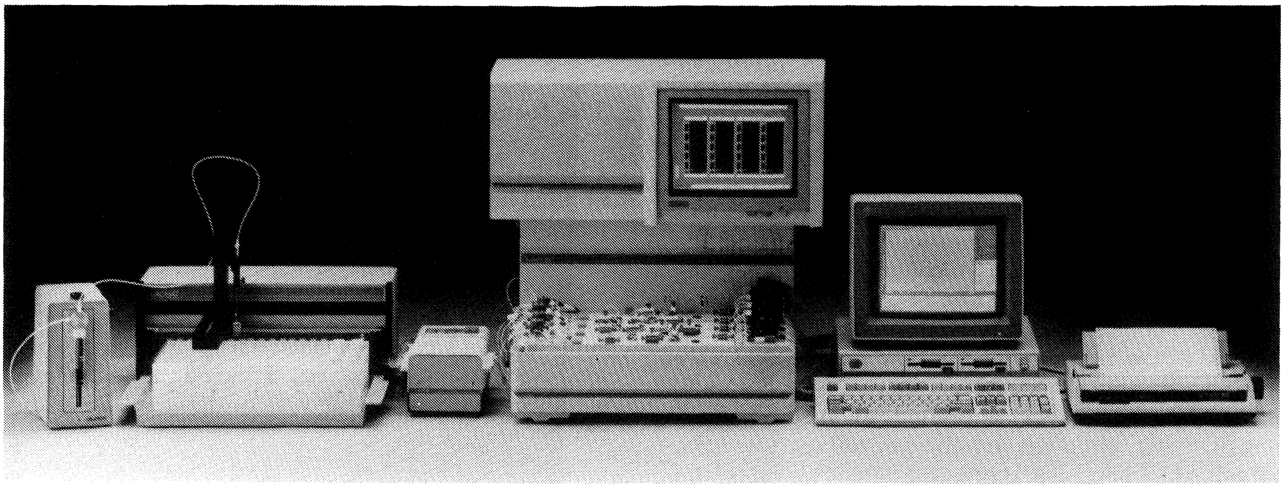

*Figure 3. Typical layout of commercially available flow injection analysis system. Instrument shown tailored to environmental markets and available from Lachat Instruments, Techmation Building, 20 Quai de la Matne, 75019 Paris, France.*

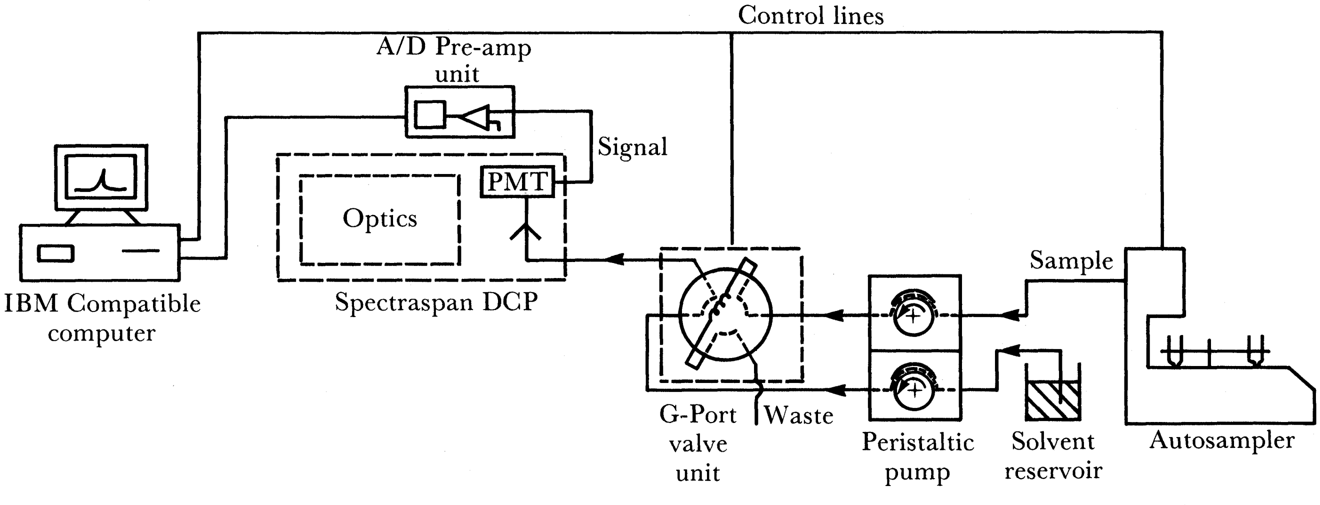

*Figure 4. Schematic diagram of the simple system designed to monitor the quality of commercial products [33].*

several excellent reviews and two monographs. The number of working routine systems is disappointingly low, due to several problem areas which are discussed below.

Basically, FIA is a type of continuous flow analysis in which the flow is not segmented with air bubbles. A quantity of dissolved sample accurately dispensed is injected or introduced into the carrier stream flowing through the FIA system. Chemical reactions or extractions which occur between the sample and the carrier can be added. As the analyte passes through the continuous detector, a transient signal is generated and recorded. The measurement is carried out under non-equilibrium conditions, since neither equilibrium, i.e. steady state or homogeneity, are realized prior to detection. This advantage of FIA can be exploited for example in process analysis. Stewart and Rosenfeld [25] have described a simple approach to measure pH. This approach gives a measurement of pH as a simple timing measurement. This is simple to do using modern microcomputer technology.

A simplistic approach to FIA analytical systems is outlined in figure 1. The liquid flow is obtained in a number of ways, most commonly using a peristaltic pump. In addition, gravity feed systems, overpressure systems on liquid vessels and simple and double piston pumps normally associated with HPLC systems, are often used. The interrelation of the pumping system and the bore size of the transport tubing to a great extent modify the theoretical considerations involved and the operation of the FIA regime. In common with CFA, the minimization of dead volumes – detector cells and between T-pieces for example – is particularly important. Injections of the sample into the flow line is invariably accomplished using six port rotary inert valves. For most FIA systems, the reaction is carried out in the normal tubing diameter, although chambers or coil reactors have been mixed. Their use, however, often obviates the principles of using FIA. The primary requirement is to use a small volume flow cell which is swept clean with minimal dead volume to provide a usable transient signal. The transient signals are characterized by the peak height or area residence or response time, or peak width at half height or at the base-line.

Where the latter is used, the overall shape of the peak is insignificant and the simple measurement of time characterizes the analytical signals. Originally, two FIA approaches were introduced – the American AMFIA System [1] which attempted to provide an automated system, and a manual unit which was developed by Ruzicka *et al.* [2]. A novel development of FIA has recently been demonstrated by Malcome-Lawes *et al.* [26] and a schematic diagram of this shown in figure 2.

### The economics of FIA

Considering the FIA approach in respect to the cost-effectiveness equations outlined above, the various time factors are significantly reduced, in comparison with both manual systems and the CFA approach. A further advantage is that the consumption of reagents and samples is reduced. Added to which, the response time, i.e. the time delay from analysis through to result generation, is substantially shortened – this means that the analyst will have a quicker and more effective response to the process being analysed or controlled. One aspect of the various equations above that is often overlooked, mainly by instrument companies, is the effect of instrument downtime and maintenance. CFA systems (which are often used in such critical areas as clinical chemistry) have become reliable due to continued improvement following user feedback. FIA systems have not been so widely accepted and so have suffered by not having this feedback.

In the context of analytical instrumentation, the marketing of the continuous flow analyser by Technicon and its consequent impact on the laboratory life is a modern day phenomenon. Stanley [27] has described the salient advantages of Technicon's approach. FIA has not had anything like the same impact. A survey of instrumentation in use in the UK by Jones [28] demonstrates this. This is in direct contrast to the evidence of the number of papers on the FIA principle which appear in the literature.

### The future of FIA

FIA has many advantages which make it a unique approach to analysis and not a substitute for existing technologies like CFA. Particularly, it uses small quantities of sample and reagents; it is a rapid analytical method; it can be simple in concept; and it allows kinetic methods and time-based systems to be used. Despite its simplicity, it needs well defined, reliable and workable instrumentation. *It is not a toy.* Figure 3 shows the layout of a commercially available system designed for a specific market area.

A major effort must be made in order to define the application areas where FIA has advantages, for example in process analysis, where the time of response is of importance to conserve product and to optimize performance. Another avenue to pursue is the development of simple specific analysis systems which can be used not in the laboratory, but at the point of sampling by untrained personnel. Finally, the use of kinetic methods and simple measurement of time as an analytical procedure offers advantages.

A recent review by Valcárcel *et al.* [29] presents an overview of the potential application of FIA in the pharmaceutical market area. When these potential methods are in use, the future of FIA will be more firmly based.

FIA widens the options open to the automated system designer and if properly installed with well constructed and reliable instrumentation it can be a valuable asset. As soon as industrial applications are found in routine use then FIA will be translated from 'novelty' value units, to a real and reliable 'analytical tool' with many diverse applications.

Despite all this there are now significant signs that FIA is reaching some form of maturity. Several areas have been identified where it can provide a good, reliable and unique approach. For example Knapp *et al.* [30], Bysouth *et al.* [31] and Cox and McLeod [32] have used the technology to provide simple and effective sample introduction systems in atomic spectroscopy and related techniques. These often yield concentration improvements of 50 times, as well as other advantages. Some of the more sophisticated analytical instrumentation, such as that developed by Fiatron Systems Inc. (Office and Research Laboratories, 6651 N Sidney Pl., Milwaukee, Wisconsin 53209, USA), are also becoming widely used for specific applications. However, it is worth repeating that the timed advantages of the FIA are of paramount importance. Recently, Brennan *et al.* [33] have designed and developed a simple system to monitor the quality of commercial products in quality assurance departments. A schematic diagram of the system is shown in figure 4. It uses readily available commercial modules and the software developed primarily for measurements has been tailored to cope with DC Plasma signals and the customer's needs. The system has been configured to provide good quality measurements for a difficult analysis and this is a case where the FIA is the answer to the problem and not an alternative solution. This system is quickly justifying its cost. The speed of response from results can be quickly translated back to the product site, where data can be used to generate other real cost savings. In conclusion, FIA has been around for almost 15 years and is now becoming useful in the real world of analysis. With the applications shown by some recent innovations, the future of FIA in the correct environment should be extremely viable.

### References

1. Stewart, K. K., Beecher, G. R. and Hare, P. E., *Analytical Biochemistry*, **79** (1976), 162.
2. Ruzicka, J. and Hansen, E. H., *Analytica, Chimica, Acta*, **78** (1975), 31.
3. Foreman, J. K. and Stockwell, P. B., *Automatic Chemical Analysis* (Horwood, Chichester, 1975).
4. Analytical Chimica Acta, Computers and Automation, published from 1978.
5. Automatic Chemical Analysis, published continuously from 1978 to date.
6. Valcárcel, M. and Luque de Castro, M. D., *Automatic Methods of Analysis* (Elsevier, Amsterdam, 1988).
7. Stockwell, P. B. and Foreman, J. K., *Topics in Automatic Chemistry*, Vol. 1 (Horwood, Chichester, 1979).
8. Norris, K. H. and Hart, J. R., *Proceedings of the 1963 International Symposium on Humidity and Moisture*, Vol. 4 (Reinhold, New York, 1965), 19.
9. Goulden, P. D. and Brooksbank, P., *Analytical Chemistry*, **38** (1974), 1431.
10. Porter, D. G. and Dennis, A. L., *Journal of Automatic Chemistry*, **2** (1979), 134.
11. P S Analytical Ltd, Arthur House, B4 Chaucer Business Park, Watery Lane, Kemsing, Sevenoaks, Kent TN15 6QY, UK.
12. Varian Associates Ltd, 28 Manor Road, Walton on Thames, Surrey, UK.
13. Applied Research Laboratories, En Vallaire, 1024 Ecublens, Switzerland.
14. Ward, R. W. and Stockwell, P. B., *Journal of Automatic Chemistry*, **5** (1983), 193.
15. Betteridge, D., Sly, T. J., Wade, A. P. and Tillman, J. E. W., *Analytical Chemistry*, **55** (1983), 2258.
16. Wentzell, P. D., Hatton, M. J., Shiundu, P. M., Ree, R. M., Wade, A. P., Betteridge, J. and Sly, T. J., *Journal of Automatic Chemistry*, **11** (1989), 227.
17. Carter, J. M. and Nickless, G., *Analyst*, **11** (1970), 148.
18. Jackson, C. J., Dennis, A. L., Porter, D. G. and Stockwell, P. B., *Analyst*, **103** (1978), 317.
19. Stockwell, P. B., *Journal of Automatic Chemistry*, **1** (1979), 216.
20. Curme, H. G., Columbus, R. L., Dappen, G. M., Elder, T. W., Fellows, W. D., Glover, C. P., Gotte, C. A., Hill, D. E., Lawton, W. H., Muta, E. J., Rand, J. E., Sandford, K. J. and Wu, T. W., *Clinical Chemistry*, **24** (1978), 1335.
21. Spayd, R. W., Bruschi, B., Burdick, B. A., Dappen, G. M., Eikenberrry, J. N., Esders, T. W., Figueras, J., Goodhue, C. T., La Rossa, D. D., Nelson, R. W., Rand, R. N. and Wu, T. W., *Clinical Chemistry*, **24** (1978), 1343.
22. Haechel, R., Sonntag, O. and Petry, K., *Journal of Automatic Chemistry*, **1** (1979), 273.
23. Clark, P. M. S. and Broughton, P. M. G., *Journal of Automatic Chemistry*, **5** (1983), 27.
24. Rujicka, J., *Analytical Chemistry*, **55** (1983), 1040A.
25. Stewart, K. K. and Rosenfeld, A. G., *Analytical Chemistry*, **54** (1982), 2368.
26. Malcome-Lawes, D. J., Milligan, G. A. and Newton, R., *Journal of Automatic Chemistry*, **9** (1987), 179.
27. Stanley, R. J., *Journal of Automatic Chemistry*, **6** (1984), 175.
28. Jones, K., *Journal of Automatic Chemistry*, **6** (1984), 175.
29. Valcárcel, M., Rios, A. and Luque de Castro, M. D., *Journal of Pharmaceutical and Biomedical Analysis*, **3** (1985), 105.
30. Knapp, G., Müller, K., Strunz, M. and Wegscheider, W., *Journal of Analytical Atomic Spectroscopy*, **2** (1987), 611.
31. Bysouth, S. R., Tyson, J. F. and Stockwell, P. B., *Analytica Chimica Acta*, **214** (1988), 329.
32. Cox, A. G. and McLeod, C. W., *Analytica Chimica Acta*, **179** (1986), 487.
33. Brennan, M. C., Svehla, G., Simons, R. A. and Stockwell, P. B., *Journal of Automatic Chemistry* (1990) (in press).

